# A rare case report of giant de novo cervical cystic hygroma in an elderly patient: Case report

**DOI:** 10.1016/j.amsu.2021.102210

**Published:** 2021-03-04

**Authors:** Salah Hammed, Areeg Al Assaf, Ali Hammed, Ziad Saker, Ali Dway

**Affiliations:** a Faculty of Medicine, Aleppo, Syria; b Almouwasat University Hospital, Otolaryngology-Head and Neck Surgery, Damascus, Syria; c Tishreen University Hospital, Department of Neurosurgery, Lattakia, Syria; d Faculty of Medicine, Tartus, Syria; e Faculty of Medicine, Lattakia, Syria

**Keywords:** Cystic hygroma, Cervical mass, Lymphangioma

## Abstract

**Introduction and importance:**

Cystic hygroma is an aberrant proliferation of lymphatic vessels resulting from abnormal development of the lymphatic system.

Cervical lymphangioma is an uncommon entity, usually reported in children, rarely in adult.

**Case presentation:**

Our case presents a 65-year-old man with an extremely huge right-sided painless cervical swelling since 8 years with no symptoms.

**Clinical discussion:**

Computer tomography (CT) showed a cystic lesion with thin walls of 150 × 100mm, medial to right sternocleidomastoid muscle (SCM).

The lesion was completely resected along with the entire capsule.

Post-operatively, the patient recovered well with no signs of any neurological dysfunction and he was discharged from hospital after 2 days.

**Conclusion:**

The etiology in the adult population is controversial.

Diagnosis in adults is considered to present a greater challenge than in children, and final diagnosis is usually based on postoperative histology.

Cystic hygroma should be considered as a differential diagnosis of cervical masses in adults.

## 1 Introduction

Cystic hygroma is an aberrant proliferation of lymphatic vessels resulting from abnormal development of the lymphatic system [1,2]. Within the literature the term cystic hygroma is used interchangeably with lymphangioma and lymphatic malformation [1,2].

Cystic hygroma also known as water-tumor or lymphangioma is a benign malformation of lymphatic vessels which usually occurs when the lymphatic system fails to communicate with the normal jugular vein. It can occur in the head, neck, axilla, cervico-facial regions, groin, and below the tongue [3].

Lymphangioma is generally known as a disease of childhood when there is active lymphatic growth. Cystic Hygromas are single or multiple cysts found mostly in the neck region. A cystic hygroma can be present as a birth defect (congenital) or develop at any time during a person's life [4].

Presentation in adulthood is rare, and the cause is uncertain, although trauma and upper respiratory tract infection have both been suggested as possible triggers for the onset [5].

Diagnosis in adults is considered to present a greater challenge than in children, and final diagnosis is usually based on postoperative histology.

In this case there was no identifiable cause and onset was sudden.

We want to underline the rarity of Cystic hygroma in adults and its role in the differential diagnoses for Cervical masses in adults.

Our work is a single case report and has been reported in line with the SCARE criteria [6,11].

## 2 Case presentation

A 65-year-old Man presented to our department via community referral, complained of right-sided painless cervical swelling.

Tumor onset was referred to be 8 years before it accelerated growth during recent months. There was no history of fever, trauma, toothache or pus discharge.

On examination, There was a soft painless cervical lesion of about 16 × 9 cm palpable in regions II–III-IV-V on the right side. This was classified as a stage III lesion according to the staging system proposed by de Serres(Fig. 2).

The mass was painless and the patient was asymptomatic apart from some restriction of neck movement.

The patient's review of systems and additional medical history surgical, family, psychosocial and pharmacologic were unremarkable.

**Fig. 1:**
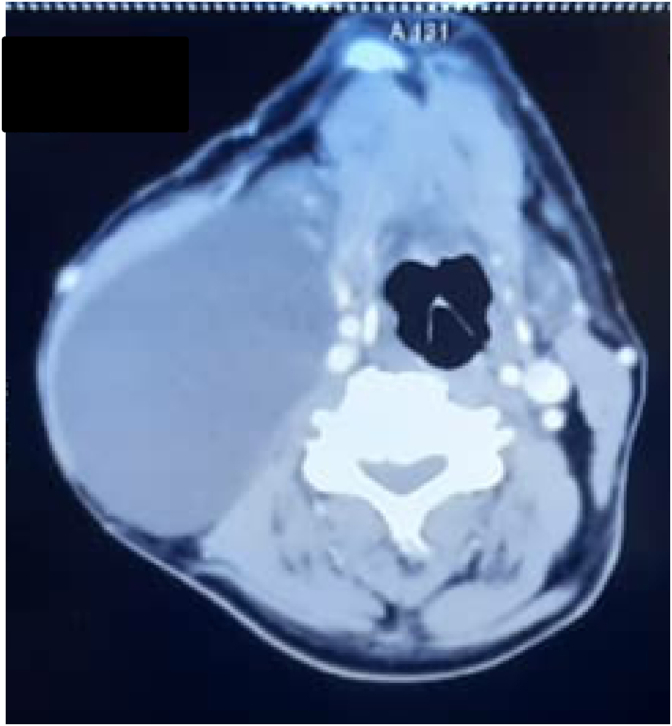
Contrast computed tomography of the neck – Axial view: Thin-walled cyst medial to the sternocleidomastoid muscle. It compresses the Carotid Sheath and is displacing the trachea and oesophagus to the left.

A contrast study of the neck using computed tomography (CT) showed a cystic lesion with thin walls of 150 × 100mm, medial to right sternocleidomastoid muscle (SCM), which compressed the Carotid Sheath and was displacing the trachea and oesophagus to the left, occupied the right II–III-IV-V levels and was clearly delimited by vascular and muscular structures(Fig. 1).

**Fig. 2:**
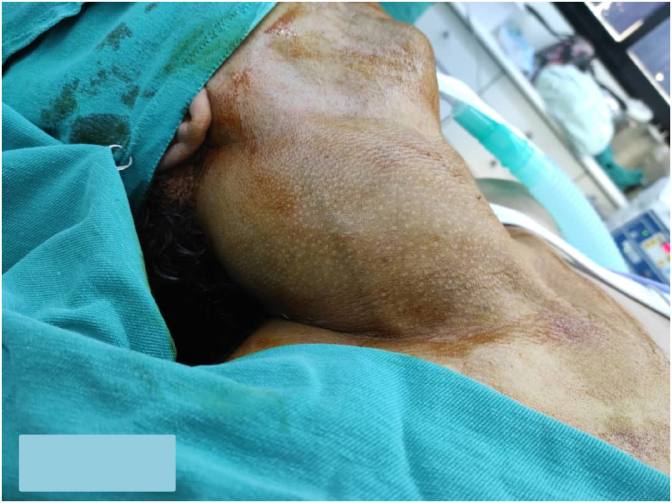
Pre-operation image shows cervical swelling extends from mastoid process to sternum on the right side.

After obtaining the patient's informed consent, surgery was planned.

The procedure was done by an otolaryngologist-head and neck surgeon.

**Fig. 3:**
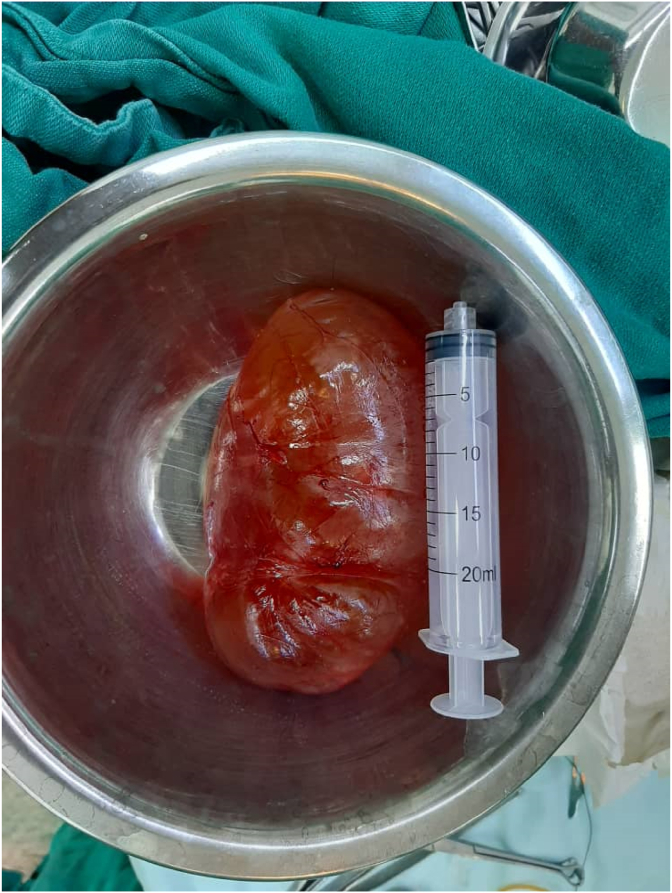
Gross feature of the mass Showing excised specimen of 15 × 10 × 8 cm in size.

The neck was explored using a reverse ‘J’ shaped incision. After retracting the SCM (sternocleidomastoid muscle) for attaining wide exposure the cyst was found abutting the IJV and carotid artery which were carefully dissected away. All the important nerves and arteries encountered during the dissection were seen and preserved. The lesion was completely resected along with the entire capsule. The measurements of the Cyst were 15 × 10 × 8 cm in size(Fig. 3).

Histological examination revealed unilocular cavity filled with uncolored fluid and lined with flat endothelial-like cells consistent with a diagnosis of cystic hygroma.

Post-operatively, the patient recovered well with no signs of any neurological dysfunction and he was discharged from hospital after 2 days.

Routine follow-up by clinical observation and ultrasound was done for past 11 months. There was no recurrence until now.

## 3 Discussion

Cystic Hygromas, also called lymphatic malformation, are anomalies of the lymphatic system characterized by single or multiple cysts within the soft tissue [7].

Lymphangioma, soft tissue tumor of disputed pathogenesis was originally reported by Reden Backer in 1828 and “cystic hygroma” name was first given by Wernker in 1834 [8].

Hygromas are probably the result of sequestration of lymphatic tissue that has retained its potential for growth in any of these areas [4].

The etiology in the adult population is controversial. Some authors attribute adult lymphangioma to delayed proliferation of the congenital or acquired lymphoid rests following trauma or preceding respiratory infections [9].

There are three histological subtype of lymphangioma's. Capillary lymphangioma (composed of small lymphatics), cavernous lymphangioma (composed of larger lymphatics), cystic lymphangioma (cystic hygroma-composed of large macroscopic lymphatic spaces with collagen and smooth muscle). Cavernous lymphangioma is the most common subtype [10].

The present case was unusual, as a large cervical cystic hygroma presented de novo in an adult with no history of trauma or upper respiratory tract infection.

They are locally aggressive, benign lesions that are difficult to manage due to recurrence of the tumor following surgery with a recurrence rate of 21% [3].

Many treatment alternatives exist for lymphangiomas, including surgical excision, laser surgery, cryotherapy, electrocautery, steroid administration, sclerotherapy, embolization and radiation therapy, but surgical excision is the most preferred option.

## 4 Conclusion

Cystic lesions of the neck are rare and difficult to interpret for clinicians, since they can be benign or malignant pathologies.

Cystic hygroma although known in pediatric age group can present at any age and should be considered as a differential diagnosis of cervical masses in adults. Surgery is the best modality of treatment and the first operative intervention offers the best opportunity for complete excision.

## Sources of funding

This research did not receive any specific grant from funding agencies in the public, commercial, or not-for-profit sectors.

## Ethical approval

This study was not applicable for ethical approval.

## Consent

I have obtained written consent for publication of this case report from the patient and I can provide this should the Editor ask to see it.

## Author's contribution

Dr. Ali Hammed (corresponding author): Contribution to the paper: data collection, data analysis and interpretation, writing the paper.

Prof Dr. Areeg Alassaf: Contribution to the paper:

Main Surgeon. Treatment and examination of the patient.

Writing Case Presentation.

Dr. Salah Hammed: Contribution to the paper: First author, Writing the paper.

Dr. Ziad Saker: Contribution to the paper: Writing the paper.

Dr.Ali dway: Contribution to the paper: Writing the paper.

## Registration of research studies

The case report at hand is not a first-in-man case report of a novel technology or surgical technique, therefore a registration of these case reports according to Declaration of Helsinki 2013 is not required.

## Guarantor

Dr. Ali Hammed.

## Provenance and peer review

Not commissioned, externally peer-reviewed.

## Declaration of competing interest

All authors declared no conflict of interest.
